# Supplementary figures and images for: Comparing lifeact and phalloidin for super-resolution imaging of actin in fixed cells
Source: PLoS One. 2021 Jan 28;16(1):e0246138. doi: 10.1371/journal.pone.0246138 (PMC7842966; doi:10.1371/journal.pone.0246138)

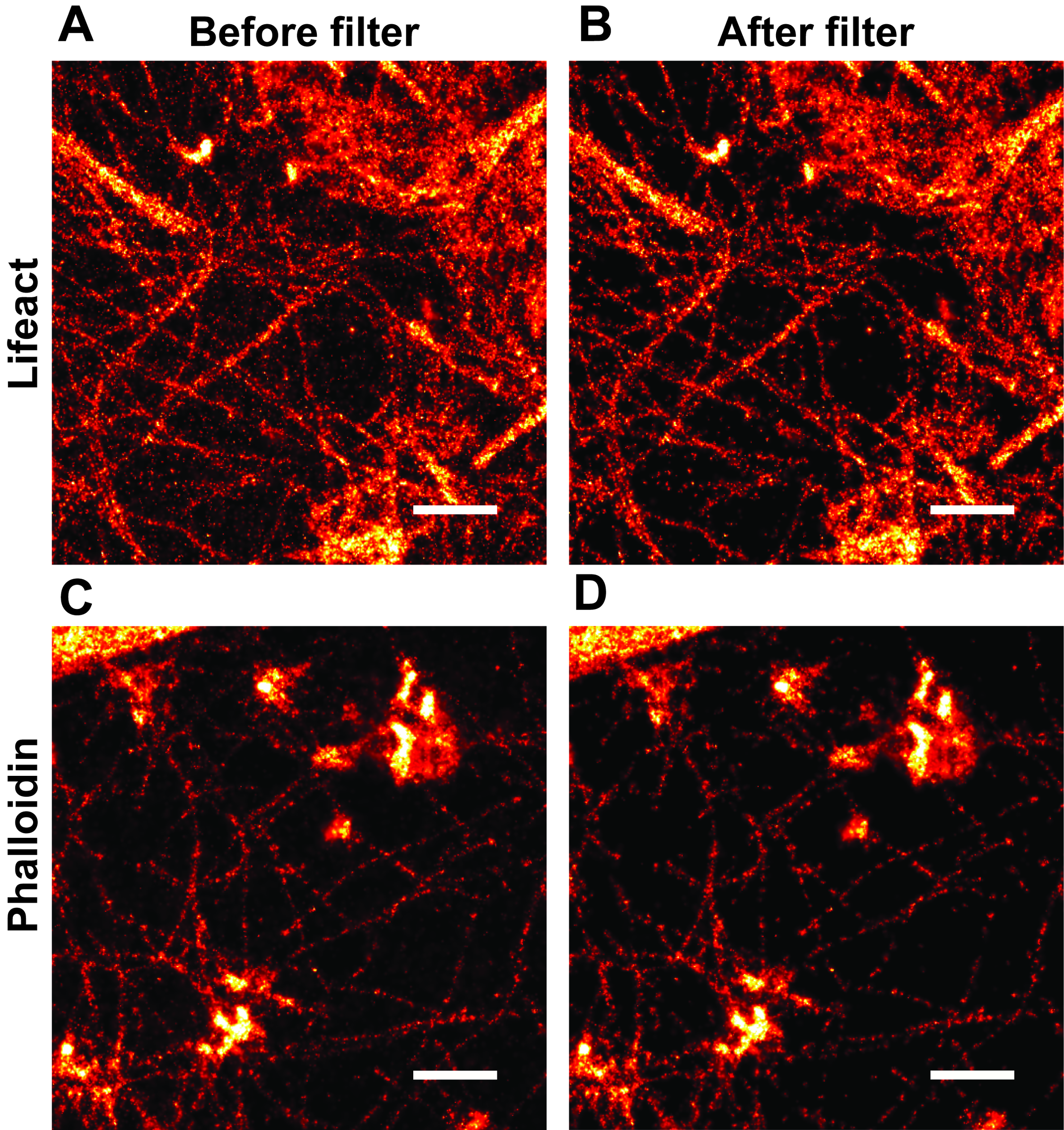

Supplement: S1 Fig — (A) Reconstructed super-resolution images of actin filaments in HeLa cells labeled with lifeact-Atto655 (top) and phalloidin-AF647 (bottom). (B) Super-resolution images of actin filaments in HeLa cells labeled with lifeact-Atto655 (top) and phalloidin-AF647 (bottom) after removing localizations that had less than 4 other localizations within 15 nm. Scale bars are 500 nm. (TIF) [file pone.0246138.s001.tif]

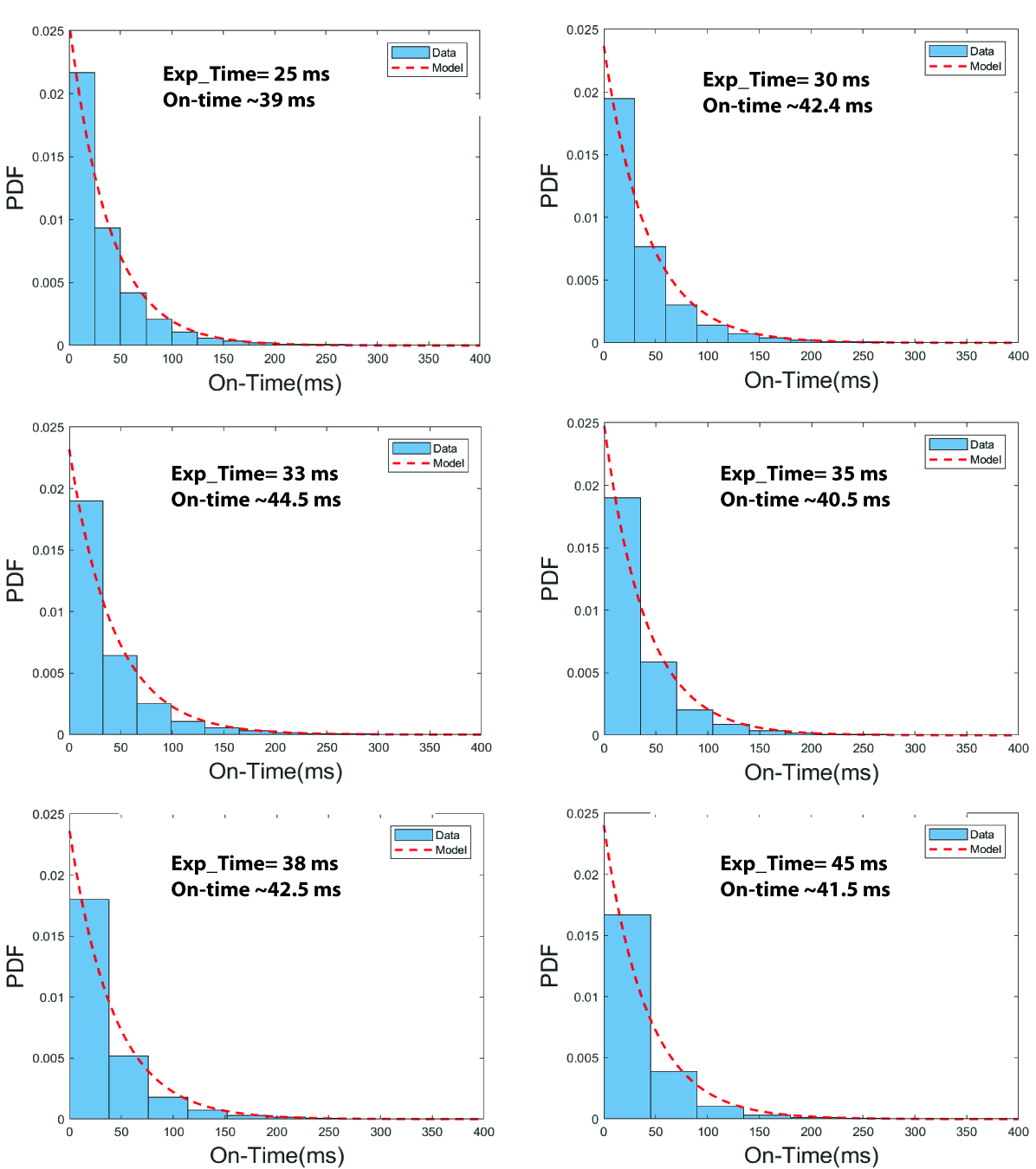

Supplement: S2 Fig — Data was collected with six exposure times: 25 ms, 30 ms, 33 ms, 35 ms, 38 ms, and 45 ms. The distribution of the number of connected localizations per binding event (blue) fits with an exponential model (red dashed line) to extract a binding lifetime. (TIF) [file pone.0246138.s002.tif]

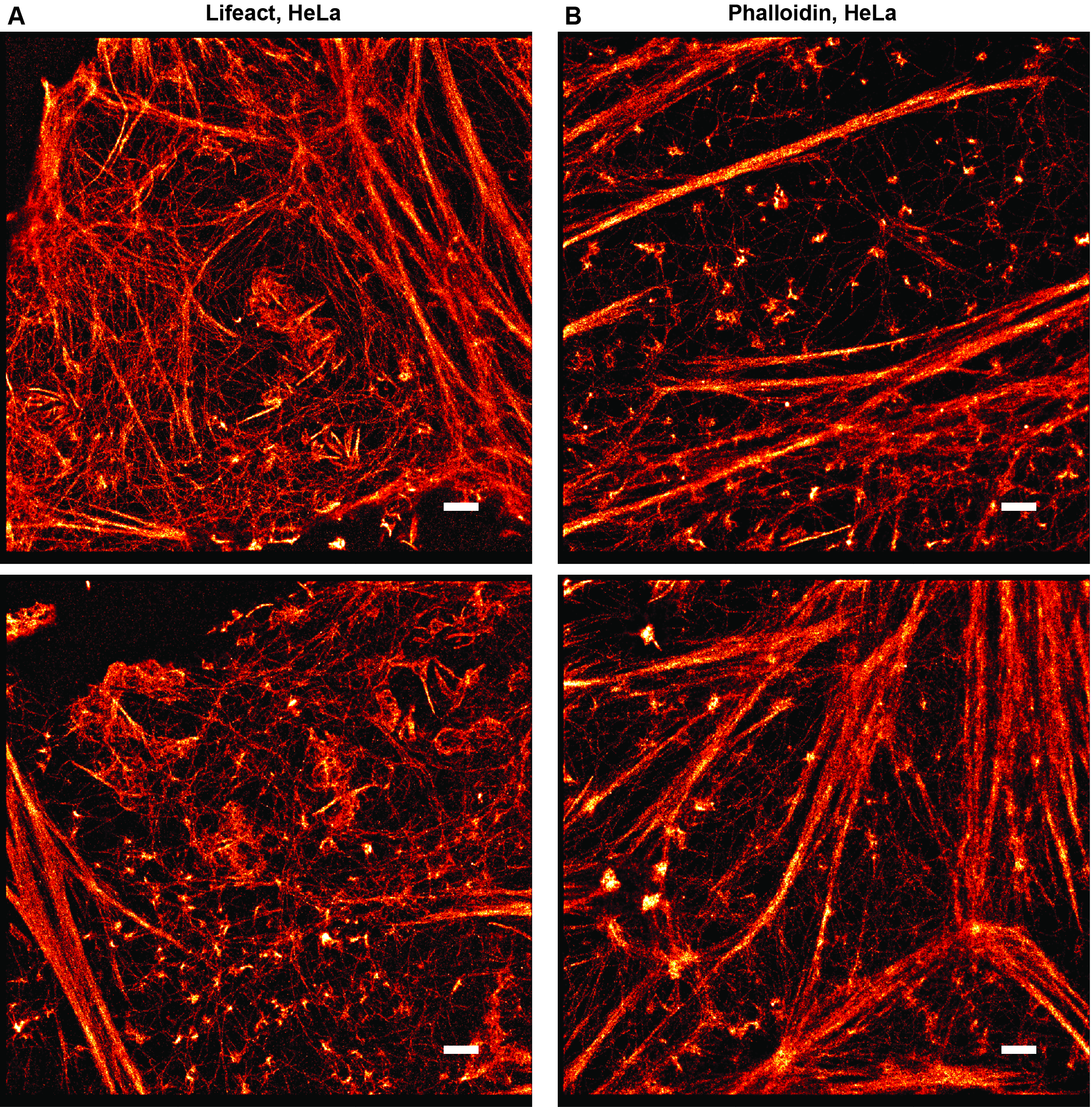

Supplement: S3 Fig — (A) Super-resolution images from two lifeact tagged samples. (B) Super-resolution image from two phalloidin tagged samples. The scale bars in full field images are 500 nm. (TIF) [file pone.0246138.s003.tif]

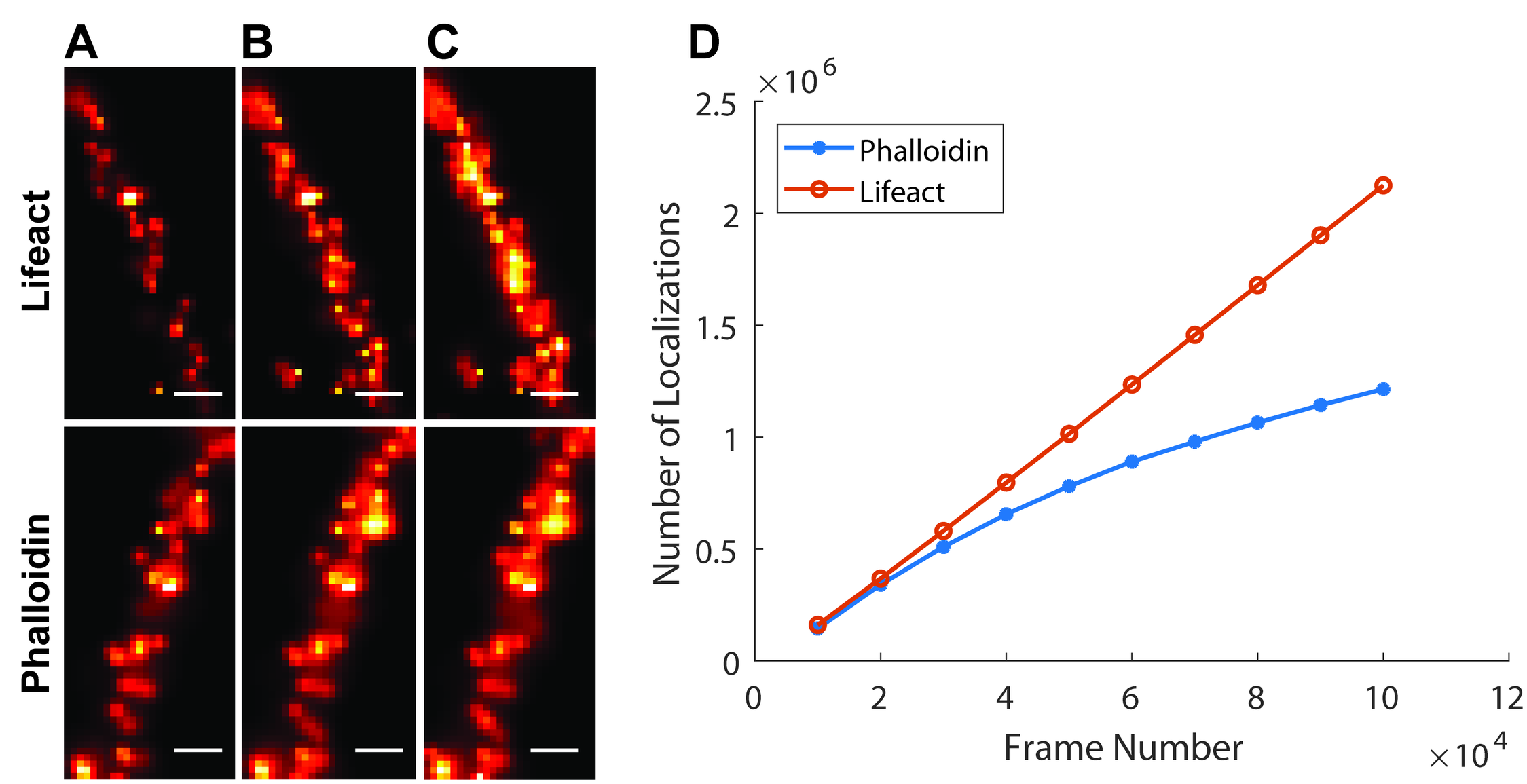

Supplement: S4 Fig — Single filaments were cropped from the reconstructed super-resolution images from lifeact-Atto655 (top) and phalloidin-AF647 (bottom). The filaments were reconstructed using localizations from the first (A) 30,000, (B) 60,000 and (C) 100,000 frames. The scale bars are 20 nm. (D) The cumulative number of localizations per frame from data collected using lifeact (red) and phalloidin (blue). Data is from HeLa cells. (TIF) [file pone.0246138.s004.tif]

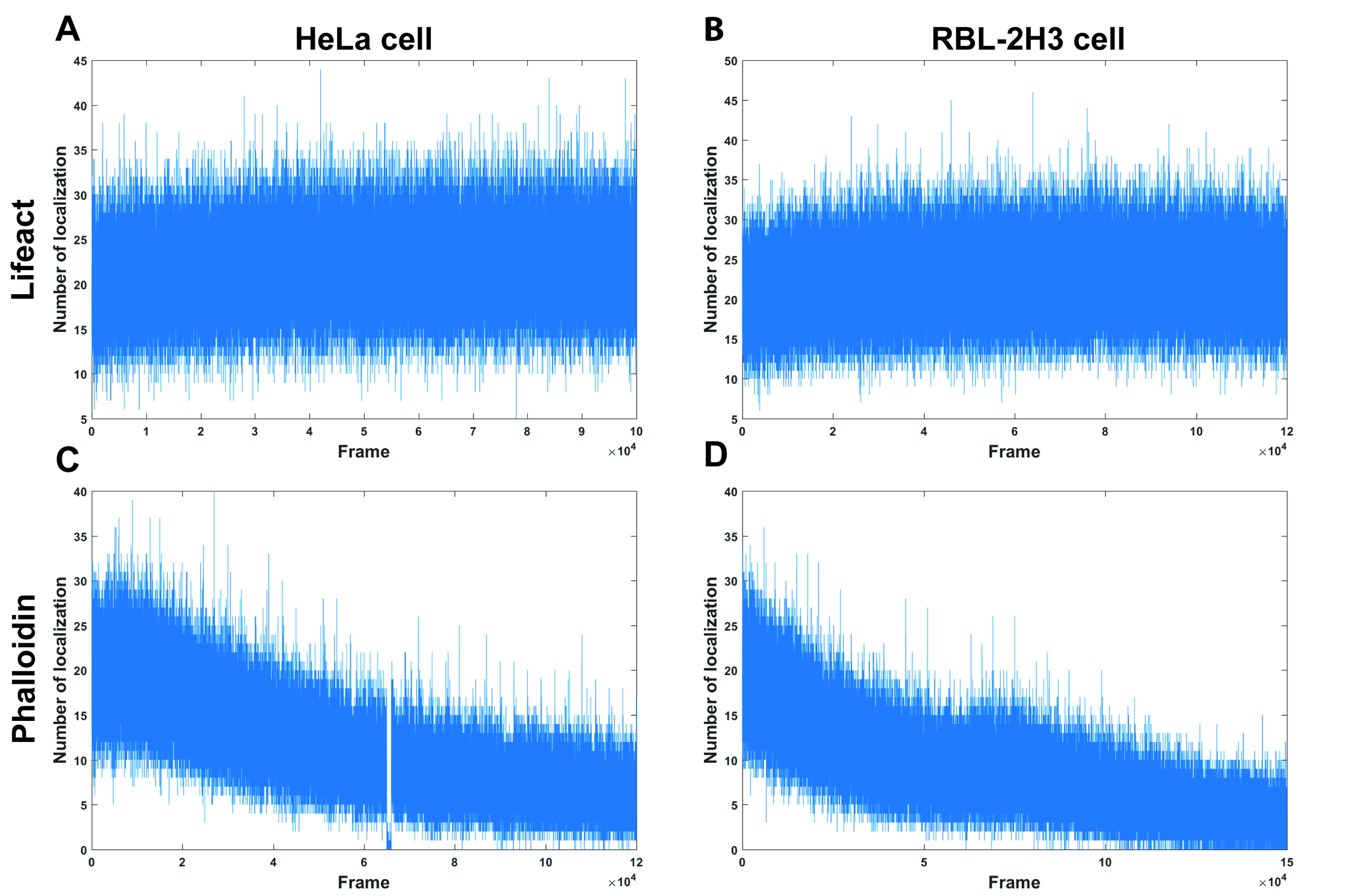

Supplement: S5 Fig — Plots show the number of localized single molecules per frame for complete data collections for individual cells. (A) HeLa cells labeled with lifeact-Atto655. (B) RBL-2H3 cells labeled with lifeact-Atto655. (C) HeLa cells labeled with phalloidin-AF647. (D) RBL-2H3 cells labeled with phalloidin-AF647. (TIF) [file pone.0246138.s005.tif]
